# Validity of the five‐item mental health inventory for screening current mood and anxiety disorders in the general population

**DOI:** 10.1002/mpr.2030

**Published:** 2024-07-02

**Authors:** Margreet Ten Have, Marja J. H. Van Bon‐Martens, Frederiek Schouten, Saskia Van Dorsselaer, Laura Shields‐Zeeman, Annemarie I. Luik

**Affiliations:** ^1^ Trimbos Institute Netherlands Institute of Mental Health and Addiction Utrecht the Netherlands; ^2^ Rijksinstituut voor Volksgezondheid en Milieu (RIVM) Bilthoven the Netherlands; ^3^ Department of Interdisciplinary Social Science Utrecht University Utrecht the Netherlands; ^4^ Department of Epidemiology Erasmus MC University Medical Center Rotterdam the Netherlands

**Keywords:** anxiety disorders, general population, mood disorders, screening, sensitivity, specificity

## Abstract

**Objectives:**

The Mental Health Inventory (MHI‐5) is frequently used as a screener for mood and anxiety disorders. However, few population‐based studies have validated it against a diagnostic instrument assessing disorders following current diagnostic criteria.

**Methods:**

Within the third Netherlands Mental Health Survey and Incidence Study (NEMESIS‐3), a representative population‐based study of adults (*N* = 6194; age: 18–75 years), the MHI‐5 was used to measure general mental ill‐health in the past month. Presence of mood (major depressive disorder, persistent depressive disorder, or bipolar disorder) and anxiety disorders (panic disorder, agoraphobia, social phobia, or generalized anxiety disorder) in the past month was assessed with a slightly modified version of the Composite International Diagnostic Interview 3.0 per the Diagnostic and Statistical Manual of Mental disorders‐5.

**Results:**

The MHI‐5 was good to excellent at distinguishing people with and without a mood disorder, an anxiety disorder, and any mood or anxiety disorder. The cut‐off value associated with the highest sensitivity and highest specificity for mood disorder was ≤68, and ≤76 for an anxiety disorder or any mood or anxiety disorder.

**Conclusions:**

The MHI‐5 can identify individuals at high risk of a current mood or anxiety disorder in the general population when diagnostic interviews are too time consuming.

## 1 INTRODUCTION

To continue to improve the mental health of the population, policymakers often need up‐to‐date information about the mental health of the population. Usually, up‐to‐date data on the prevalence of common mental disorders in the population is scarce, as medical registries and insurance records only cover those who sought help, and large‐scale representative epidemiological studies that use specially designed instruments to assess mental disorders are limited. Decisions may therefore be based on available data, using more readily available data captured through screeners for mental ill‐health.

Many screeners and questionnaires for measuring mental ill‐health are available. Some of the most frequently used short screeners (<10 items) for mental ill‐health only screen for a single mental disorder, such as the 9‐item Patient Health Questionnaire (PHQ‐9: Kroenke et al., 2001) which measures depressive symptoms and the 7‐item Generalized Anxiety Disorder Assessment (GAD‐7: Spitzer et al., 2006) which measures symptoms of anxiety and worry. The five‐item Mental Health Inventory (MHI‐5; Ware & Sherbourne, 1992) and the six‐item Kessler Screening Scale (K6: Kessler et al., 2003) are examples of questionnaires that assess a broader concept of mental health, where the MHI‐5 is more commonly used than the K6, not only in surveys of mental health but also in surveys of general health and quality of life (Berwick et al., 1991; Hoeymans et al., 2004). The MHI‐5 is short and easy to administer, and has proven to be a valid screener of general mental health in previous research (Cuijpers et al., 2009). Therefore, the MHI‐5 is often the questionnaire of choice for obtaining an up‐to‐date estimate of mental health of the general population.

Research comparing the performance of the MHI‐5 with an instrument designed to diagnose mental disorders has shown that the MHI‐5 could be a valid instrument for detecting mood or anxiety disorders in the general population (Batterham et al., 2018; Cuijpers et al., 2009; Rumpf et al., 2001), as it is for detecting major depression in two specific groups, that is, functionally impaired, community‐dwelling elderly (Friedman et al., 2005) and seropositive individuals (Holmes, 1998). The MHI‐5 also performed well in distinguishing mood and anxiety disorders in patients (Berwick et al., 1991), and between patients with and without a clinical psychiatric history (Santos & Novo, 2019). In general, the MHI‐5 is more accurate in detecting mood disorders compared to anxiety disorders (Berwick et al., 1991; Cuijpers et al., 2009; Rumpf et al., 2001) due to the items selected for this short screener.

Of the studies available, only two validation studies were based on a representative sample from the general population (Cuijpers et al., 2009) or region (Rumpf et al., 2001). Yet, these were performed in the late 1990s and have used clinical instruments based on the Diagnostic and Statistical Manual of Mental Disorders (DSM)‐III‐R and DSM‐IV. Although changes have been relatively small for mood and anxiety disorders compared to some other disorders, this may affect the relevance of these studies today. Other studies were based on specific groups (Friedman et al., 2005; Holmes, 1998) and patients (Berwick et al., 1991), also based on older diagnostic criteria. Only one recent study compared the MHI‐5 to DSM‐5 diagnostic criteria but the study employed a selective sample from the general population and used an online symptom checklist instead of a clinical interview (Batterham et al., 2018).

The present study attempts to leverage a nationally representative population based study to assess the accuracy of the MHI‐5 in detecting DSM‐5 mood and anxiety disorders in a general adult population, using a structured clinical interview as the “gold standard” for diagnoses. Specifically, we analyzed whether the MHI‐5 is a good screener for mood and anxiety disorders assessed in our third Netherlands Mental Health Survey and Incidence Study (NEMESIS‐3) and determined optimal cut‐off points that could be used in future research to estimate general mental health in the adult population.

## 2 METHOD

### 2.1 Study design

NEMESIS‐3 is based on a multistage, stratified random sampling procedure. First, a random sample of municipalities was drawn. Second, a random sample of individuals aged 18–75 years was drawn from the Dutch population register. Individuals with insufficient command of the Dutch language, as well as institutionalized individuals (i.e., those living in hostels, hospices or prisons), were excluded. Individuals temporarily living in institutions were contacted to be interviewed after returning home.

For NEMESIS‐3, no official ethical approval on how to perform the study was required under the Dutch Medical Research Involving Human Subjects Act (WMO; reference number: WAG/mb/19/017126; May 15, 2019). The study proposal, field procedures, information for respondents, and informed consent forms were assessed positively by a local ethical review committee. Respondents provided written informed consent to participate in the interview, after full written and verbal information about the study was given before and at the start of the interview.

The interviews were laptop computer‐assisted, and almost all were held at the respondent's home. In the first wave, performed from November 2019 to March 2022, 6194 persons were interviewed (response rate: 54.6%; average interview duration: 91 min). Respondents reflected the Dutch population reasonably well. Younger people, higher secondary educated people, those not living with a partner, people living in bigger towns, and people of non‐Dutch origin were somewhat underrepresented. A comprehensive description of the design and results of the first wave of NEMESIS‐3 can be found elsewhere (Ten Have, Tuithof, van Dorsselaer, Schouten, & de Graaf, 2023, Ten Have, Tuithof, van Dorsselaer, Schouten, de Graaf, & Luik, 2023).

### 2.2 Measures

The MHI‐5 is a sub‐scale of the 36‐item Short Form Health Survey (SF‐36; Ware & Sherbourne, 1992), and consists of five items that ask respondents how much of the time in the past four weeks they had considered themselves to: (1) feel very nervous; (2) felt so down in the dumps that nothing could cheer them up; (3) felt calm and relaxed; (4) felt depressed and sad; (5) felt happy. The answers are scored on six‐point scales ranging from all of the time to none of the time. The total score is calculated by reversing the answers to two items (the third and fifth), summing up the scores, and transforming the raw scores to a scale ranging from zero to 100. A higher score indicates better mental health. The MHI‐5 was administered after the Composite International Diagnostic Interview (CIDI) during the face‐to‐face interviews. The reliability of the MHI‐5 (Cronbach's alpha) in the first wave was 0.80 (with 95% CI: 0.79–0.81).

A slightly modified CIDI version 3.0 was used to enable both Diagnostic and Statistical Manual of Mental Disorders‐IV (DSM‐IV) and DSM‐5 disorders (Ten Have, Tuithof, van Dorsselaer, Schouten, & de Graaf, 2023). The CIDI 3.0 is a fully structured diagnostic interview, developed for use in the World Mental Health Survey Initiative (Kessler & Ustün, 2004), and assesses DSM‐IV mood and anxiety disorders with generally good validity (Haro et al., 2006). Although the criteria for these disorders according to DSM‐IV and DSM‐5 are quite similar, the validity and reliability of our modified CIDI 3.0 to assess DSM‐5 mood and anxiety diagnoses have not been investigated.

For the current study, we used the diagnoses of mood disorders (Major Depressive Disorder (MDD), Persistent depressive disorder (PDD), bipolar disorder) and anxiety disorders (panic disorder, agoraphobia, social anxiety disorder or social phobia, specific phobia, generalized anxiety disorder (GAD)). Most of the DSM‐5 diagnostic criteria for these mental disorders were assessed within the CIDI, with the exception of two criteria: symptoms not attributable to substance use or medication and not better explained by a schizophrenia spectrum and psychotic disorder. Additionally, the symptoms may be due to a condition we did not assess in our study, such as obsessive‐compulsive disorder (OCD) and posttraumatic stress disorder.

Because we are interested in the association between the MHI‐5, which assesses symptoms over the past 4 weeks, and current mood and anxiety disorders, we used diagnoses over the past month. According to the algorithms of the CIDI 3.0, someone has a 1‐month disorder if the symptoms meet the criteria for a 12‐month disorder and if the core symptoms are still present in the past 4 weeks. Examples of core symptoms considered are sadness or anhedonia, along with other symptoms of depression discussed earlier in the interview, that have lasted 2 weeks or more in the past 4 weeks for MDD, and a period of excessive anxiety and worry that had lasted 1 month or more in the past 4 weeks for GAD.

### 2.3 Statistical analyses

We first calculated the means and standard deviations of the MHI‐5 for people with each of the mood and anxiety disorders separately, and also for people with single disorders and those with the most frequent combinations of disorders. Then we performed a Receiver Operating Characteristic (ROC) analyses and examined the Area Under the Curve (AUC) for the MHI‐5 with each of the mood and anxiety disorders as a gold standard. An AUC of <0.70 is considered poor, an AUC between ≤ 0.70 and < 0.80 acceptable, an AUC between ≤0.80 and < 0.90 excellent, and an AUC ≥ 0.90 outstanding in correctly distinguishing between people with and without a disorder (Hosmer et al., 2013). Based on these criteria, we determined to what extent we could include individual disorders in the overarching main group of disorders (i.e., either mood or anxiety disorders) and in the definitions of any mood or anxiety disorder and at least two mood or anxiety disorders. Based on a low AUC for specific phobia (AUC: 0.64, 95% Confidence Interval (CI): 0.61–0.67), we excluded this disorder from the category anxiety disorders.

We then calculated the optimal cut‐off points on the MHI‐5 for four main groups of disorders (i.e., a mood disorder, an anxiety disorder, any mood or anxiety disorder, multiple mood or anxiety disorders), using two different methods (the Euclidian method and the Youden method), as there is no clear consensus on which method is most suitable (Hajian‐Tilaki, 2018) and to have an indication of the consistency of the cut‐off between methods. Of the two methods, the Youden method is preferred, as it has been used more frequently in prior studies and therefore facilitates greater comparability with other studies. In the Youden method, the optimal cut‐off is determined by the highest sum of the sensitivity and specificity‐1. The Euclidian method sets the point closest to the 0,1 corner on the ROC curve as the optimal cut‐off point (Hajian‐Tilaki, 2018; Kelly et al., 2008).

To assess the performance of the determined cut‐offs for specific disorders, we calculated the sensitivity and specificity of these cut‐off values on the MHI‐5 for their respective specific disorders, that is, cut‐off value for any mood disorder for the specific mood disorders, cut‐off value for any anxiety disorder for the specific anxiety disorders, cut‐off value for at least two mood or anxiety disorders for the most common combinations of disorders.

We then applied the optimal cut‐off points for the main groups of disorders to eight different subpopulations (males, females, and six age groups) and calculated the associated sensitivity and specificity for these subgroups to assess the performance of the established cut‐offs in these groups.

Analyses were based on all people with complete data on both mood and anxiety disorders and the MHI‐5 (*n* = 6186). The analyses were conducted with the statistics program R 4.2.2 (R Core Team, 2022, https://www.r‐project.org/), using the pROC package (https://bmcbioinformatics.biomedcentral.com/articles/10.1186/1471‐2105‐12‐77).

## 3 RESULTS

The sample consisted of 50.4% women; 40.2% aged 55 or older; 41.4% with a higher vocational/university education, and 82.8% of Dutch origin (Table 1). According to diagnoses made using the CIDI, 2.7% met the criteria for a current mood, 5.0% for an anxiety disorder, 6.5% had any mood or anxiety disorder and 2.4% had multiple mood or anxiety disorders (Table 2).

**TABLE 1:** Baseline characteristics of the total sample (*n* = 6186), in percentages.

	%
Female gender	50.4
Age at interview	
18–24	10.8
25–34	15.1
35–44	16.2
45–54	17.7
55–64	20.4
65+	19.8
Educational level	
Primary, lower secondary	22.1
Higher secondary	36.5
Higher vocational, university	41.4
Dutch origin	82.8

**TABLE 2:** MHI‐5 scores in people with current DSM‐5 mood and anxiety disorders (1 month prevalence): means and standard deviations, AUC statistics with 95% Confidence Intervals (CI).

	*n*	%	MHI‐5			
			*M*	S.D.	AUC	95% CI
1‐month prevalence rates						
Any mood disorder	164	2.65	52.80	17.36	0.93	0.92–0.95
Major depressive disorder (MDD)^a^	139	2.25	52.86	17.29	0.93	0.92–0.95
Persistent depressive disorder (PDD)^b^	77	1.24	50.03	18.08	0.94	0.91–0.96
Bipolar disorder^c^	22	0.36	52.55	17.90	0.92	0.86–0.97
Single disorders						
Any mood disorder only	95	1.54	58.82	14.78	0.90	0.88–0.93
MDD only	41	0.66	59.71	15.98	0.89	0.85–0.93
Any anxiety disorder^d^	308	4.98	62.83	19.26	0.84	0.82–0.86
Panic disorder	50	0.81	59.20	22.61	0.82	0.76–0.89
Agoraphobia (AG)	66	1.07	57.88	20.24	0.87	0.83–0.91
Social phobia (SO)	186	3.01	64.73	18.69	0.82	0.78–0.85
Generalized anxiety disorder (GAD)	97	1.57	52.41	17.81	0.93	0.90–0.95
Single disorders						
Any anxiety disorder only^d^	239	3.86	68.12	16.35	0.80	0.77–0.83
SO only	119	1.92	71.73	15.59	0.75	0.70–0.79
GAD only	36	0.58	60.22	13.62	0.89	0.84–0.94
Any mood or anxiety disorder^d^	403	6.51	61.89	18.36	0.86	0.84–0.88
At least two mood or anxiety disorders^d^	147	2.38	51.65	17.30	0.94	0.92–0.96
Comorbid disorders						
MDD + PDD	74	1.20	49.95	18.01	0.94	0.91–0.96
MDD + GAD	35	0.57	42.74	19.42	0.96	0.92–0.99
SO + AG	33	0.53	51.88	19.79	0.91	0.87–0.96
SO + specific phobia	45	0.73	56.00	18.78	0.89	0.85–0.94
GAD + PDD	24	0.39	41.83	21.62	0.95	0.90–0.99
GAD + SO	33	0.53	48.61	15.94	0.95	0.93–0.97

^a^ MDD includes the DSM‐codes 296.21, 296.22, 296.23, 296.31, 296.32, and 296.33.

^b^ PDD includes the DSM‐code 300.4.

^c^ Bipolar disorder includes the DSM‐codes 296.41, 296.42, 296.43, 296.51, 296.52, 296.53, and 296.89.

^d^ Specific phobia was not included in the category of anxiety disorders as the AUC was poor (AUC: 0.64, 95% Confidence Interval (CI): 0.61–0.67).

People with a mood disorder had a lower mean score on the MHI‐5, indicating poorer mental health, compared to those with an anxiety disorder (Table 2: 52.8 vs. 62.8). As a result, people with any mood or anxiety disorder had an average score on the MHI‐5 between these values: 61.9. The more mood or anxiety disorders people had, the lower they scored on the MHI‐5 (85.6, 67.8 and 51.7 for those with 0, 1 and 2 diagnoses, respectively). People with a single disorder had a higher MHI‐5 score compared to those with a comorbid disorder: its mean varied between 58.8 (mood disorder only) and 71.7 (social phobia only). People with comorbid disorders had a mean score on the MHI‐5 that ranged between 41.8 (comorbidity of GAD and PDD) and 56.0 (comorbidity of social and specific phobia).

The AUC of the MHI‐5 for a mood disorder, which indicates the ability to detect people with and without any mood disorder based on MHI‐5 scores, was outstanding (0.93). For an anxiety disorder and for distinguishing any mood or anxiety disorder, it was somewhat lower, but still excellent (0.84 and 0.86, respectively). The AUC for detecting people with multiple mood or anxiety disorders compared to those with one or no disorder was outstanding (0.93). The AUCs of the MHI‐5 for the single disorders were mostly excellent, except for the AUC of social phobia only which was acceptable. Those for different combinations of comorbid disorder were excellent to outstanding.

When comparing persons with a 1‐month disorder (Table 2) and a 12‐month disorder (Table S1), those with a 1‐month disorder had lower mean scores on the MHI‐5 than those with a 12‐month disorder. The AUCs of the MHI‐5 for 1‐month disorders and disorder patterns were better than those for 12‐month disorders and disorder patterns, except for the comorbidity pattern of social phobia and agoraphobia where the AUCs for a 1‐month and 12‐month disorder pattern were similar.

Both methods to calculate the optimal cut‐off scores on the MHI‐5 resulted in the same scores for a mood disorder (both ≤68), an anxiety disorder (both ≤76), and any mood or anxiety disorder (both ≤76). The cut‐off value on the MHI‐5 for a mood disorder was associated with a sensitivity of 0.84 and a specificity of 0.88 (Table 3). This means that it would correctly assign 84% with a mood disorder and 88% without a mood disorder. The cut‐off values for an anxiety disorder and for any mood or anxiety disorder resulted in somewhat lower but still acceptable sensitivity and specificity values (0.76 and 0.78, and 0.79 and 0.79, respectively). The optimal cut‐off score on the MHI‐5 for multiple mood or anxiety disorders was ≤72 according to Youden's method, and ≤68 according to Euclidean's method. The first method resulted in a higher sensitivity but lower specificity compared to the Euclidean's method (0.92 and 0.83, and 0.85 and 0.88, respectively).

**TABLE 3:** Optimal cut‐off scores of the MHI‐5 for any mood disorder (≤68), for any anxiety disorder (≤76), and for any mood or anxiety disorder (≤76) and associated sensitivity and specificity measures for current DSM‐5 mood disorders, anxiety disorders and a combination of these disorders, respectively.

	MHI‐5 cut‐off score	Sensitivity	Specificity
1‐month prevalence rates			
Any mood disorder	≤68	0.84	0.88
Major depressive disorder (MDD)	≤68	0.85	0.88
Persistent depressive disorder (PDD)	≤68	0.90	0.87
Bipolar disorder	≤68	0.77	0.87
Single disorders			
Any mood disorder only	≤68	0.79	0.87
MDD only	≤68	0.71	0.87
Any anxiety disorder^a^	≤76	0.76	0.78
Panic disorder	≤76	0.70	0.76
Agoraphobia (AG)	≤76	0.79	0.76
Social phobia (SO)	≤76	0.76	0.77
Generalized anxiety disorder (GAD)	≤76	0.94	0.76
Single disorders			
Any anxiety disorder only^a^	≤76	0.69	0.77
SO only	≤76	0.65	0.76
GAD only	≤76	0.89	0.76
Any mood or anxiety disorder^a^	≤76	0.79	0.79
Comorbid disorders^a^	≤72	0.92	0.83
MDD + PDD	≤72	0.95	0.82
MDD + GAD	≤72	0.97	0.82
SO + AG	≤72	0.91	0.82
SO + specific phobia	≤72	0.82	0.82
GAD + PDD	≤72	0.96	0.82
GAD + SO	≤72	0.94	0.82

^a^ Specific phobia was not included in the category of anxiety disorders as the optimal cut‐off score on the MHI‐5 for this disorder was associated with a very low sensitivity (0.65) and specificity (0.56).

The determined cut‐off values showed acceptable to excellent sensitivity and specificity values for the specific mood disorders and anxiety disorders, with the exception of “any anxiety disorder only” and “social phobia only” (Table 3). The cut‐off value for at least two mood or anxiety disorders also resulted in excellent sensitivity and specificity values for the most common combinations of disorders.

When we applied the optimal cut‐off value for a mood disorder to the different subpopulations (males, females, six age groups), we found acceptable sensitivity and specificity values (all ≥0.70, see Table 4). Sensitivity varied between 0.70 and 0.93, and specificity between 0.84 and 0.91. The optimal cut‐off value for an anxiety disorder also resulted in acceptable sensitivity and specificity values for the different subpopulations, except for those aged 65 and over. In this age group, a cut‐off score of ≤76 on the MHI‐5 for an anxiety disorder was associated with a very low sensitivity of 0.59 and an acceptable specificity of 0.81. The optimal cut‐off value for any mood or anxiety disorder based on the total population also performed well across all subpopulations. The associated sensitivity values varied between 0.73 and 0.85, and specificity values between 0.73 and 0.83. The optimal cut‐off value for multiple mood or anxiety disorders according to Youden's method showed similar or even better results: the cut‐off value of ≤72 resulted in sensitivity values between 0.82 and 1.00 and specificity values between 0.78 and 0.87.

**TABLE 4:** Cut‐off scores of the MHI‐5 and associated sensitivity and specificity measures for main categories of current mood and anxiety disorder and for current multiple mood or anxiety disorders.

	Any mood disorder (cut‐off ≤68)		Any anxiety disorder (cut‐off ≤76)		Any mood or anxiety disorder (cut‐off ≤76)		At least two mood or anxiety disorders (cut‐off ≤72)	
	Sensitivity	Specificity	Sensitivity	Specificity	Sensitivity	Specificity	Sensitivity	Specificity
Total population	0.84	0.88	0.76	0.78	0.79	0.79	0.92	0.83
Males	0.78	0.91	0.71	0.82	0.76	0.83	0.84	0.87
Females	0.87	0.86	0.78	0.74	0.81	0.75	0.94	0.80
18–24	0.70	0.87	0.84	0.73	0.82	0.75	0.91	0.79
25–34	0.93	0.84	0.71	0.72	0.75	0.73	0.93	0.78
35–44	0.91	0.88	0.80	0.78	0.84	0.79	0.88	0.84
45–54	0.86	0.89	0.83	0.79	0.85	0.80	1.00	0.84
55–64	0.77	0.90	0.72	0.81	0.75	0.82	0.82	0.85
65	0.85	0.90	0.59	0.81	0.73	0.82	1.00	0.86

## 4 DISCUSSION

### 4.1 Key findings

The MHI‐5 is a useful and very suitable tool for screening for mood disorders (MDD, PDD, bipolar disorder) and anxiety disorders (panic disorder, agoraphobia, social phobia, GAD) in the past month in the general adult population. Properties for discriminating those with mood disorders seem slightly better than those with anxiety disorders. The AUC for any mood or anxiety disorder was also adequate. The cut‐off value on the MHI‐5 with the highest sensitivity and highest specificity for a mood disorder was ≤68, and for anxiety disorder and any mood or anxiety disorder it was ≤76. These cut‐off points generally resulted in acceptable to excellent sensitivity and specificity values when applied within subgroups differing in sex, age and specific disorders in the population, with only a few exceptions.

### 4.2 Strengths and limitations

Our study has important strengths, including the large sample of adults from the general population and the use of a diagnostic instrument to assess DSM‐5 mood and anxiety disorders. Despite these strengths, there are some limitations that should be taken into account in interpreting the findings. First, although the sample was representative of the Dutch population on most parameters, people with an insufficient mastery of Dutch, those without a permanent residence and the institutionalized were underrepresented. However, using the cut‐off values from the present study is unlikely to result in mood or anxiety problems being overlooked in in‐patients with mood or anxiety disorders.

Second, in the present study, only the most common mood and anxiety disorders according to the DSM‐5 definitions were determined and therefore other disorders were excluded, such as OCD or post‐traumatic stress disorder (PTSD), which have been considered in previous studies. In the present study, the validity of the MHI‐5 for detecting an anxiety disorder relates to one of four anxiety diagnoses. We did not include specific phobia in our definition of any anxiety disorder, because the MHI‐5 was not found to be an adequate screener for this specific anxiety disorder. Given the high comorbidity of mood and anxiety disorders with other mental disorders, we expect that we would have obtained similar cut‐off values for the main groups of disorders if we could have included less common or potentially more severe mood and anxiety disorders, like PTSD.

Third, duration and severity are not queried over the past 4 weeks per specific symptom. Rather the 1‐month diagnoses were based on meeting the criteria for a 12‐month disorder with core symptoms present in the past 4 weeks.

Fourth, it cannot be ruled out that a question‐order effect might have occurred, as the MHI‐5 was administered after the CIDI. In general, it is preferable to administer a screener before the diagnostic interview when assessing its psychometric properties (Cuijpers et al., 2009). Moreover, in the present study the MHI‐5 was part of a larger instrument; the SF‐36 (Ware & Sherbourne, 1992). This means that our findings cannot be directly translated or applied to studies that only use the MHI‐5 and not the complete questionnaire.

Fifthly, we could not test the ability of the MHI‐5 to detect disorder severity. This could be addressed in future research and is particularly relevant if one wants to know what percentage of the population has mental disorders that are associated with moderate to severe functional limitations.

### 4.3 Discussion of research findings

#### 4.3.1 Mood disorders

The excellent ability of the MHI‐5 for detecting people with and without a mood disorder, also when a single disorder without any comorbid disorder, was comparable to the AUCs for a mood disorder reported in previous population‐based studies (AUC = 0.93 vs. AUC = 0.88 in Rumpf et al., 2001 and AUC = 0.92 in Cuijpers et al., 2009). When comparing our findings to previous validation studies among primary care patients (AUC MDD = 0.89 in Berwick et al., 1991) and specific sub‐groups, such as HIV seropositive outpatients (AUC major depression = 0.84 in Holmes, 1998) and functionally impaired elderly persons (AUC major depression = 0.84 in Friedman et al., 2005), it appears that the MHI‐5 performs slightly better as a screener for MDD (AUC = 0.93) in the general population than in a less healthy population.

The optimal cut‐off value on the MHI‐5 that was associated with the highest sensitivity and highest specificity for a mood disorder was ≤68. A higher cut‐off value was previously found for MDD or dysthymia in a general population study (<74 in Cuijpers et al., 2009), whereas lower cut‐off values were previously found for a mood disorder in a regional study (<60 in Rumpf et al., 2001) and for major depression in studies among community‐dwelling elderly with functional limitations (cut‐off point of <60 in Friedman et al., 2005) and seropositive individuals (52 in Holmes, 1998). It is not surprising that in the latter two groups the values were lower, as these studies focused on settings or sub‐groups with existing health or mental health conditions, which may lead to the endorsement of symptoms based on other health problems rather than on the presence of mood or anxiety disorders.

The MHI‐5 was also excellent at detecting people with and without a specific mood disorder, such as MDD, PDD and bipolar disorder (AUC ≥ 0.92). Moreover, the optimal cut‐off value for any mood disorder resulted in acceptable to good sensitivity and specificity values for these specific mood disorders (i.e., ≥0.77).

#### 4.3.2 Anxiety disorders

In the present study, the AUC for an anxiety disorder (AUC = 0.84) was clearly higher than the AUCs reported in previous population‐based studies (AUC = 0.71 in Rumpf et al., 2001; AUC = 0.73 in Cuijpers et al., 2009) and in a study among patients (AUC = 0.74 in Berwick et al., 1991). This may be partly explained by the fact that we excluded specific phobias from our definition of any anxiety disorder, as the MHI‐5 was not an adequate screener for this disorder (AUC = 0.64), something that has also been found in prior research (Cuijpers et al., 2009). A possible explanation for this is that people with specific phobias do not recognize their anxiety and avoidance behavior in the generalized anxiety symptoms assessed in the MHI‐5. Additionally, previous studies typically included OCD within the anxiety disorders, as it was included as an anxiety disorder in the DSM‐IV and DSM‐III (Berwick et al., 1991; Cuijpers et al., 2009; Rumpf et al., 2001). Within these studies, only Cuijpers et al. (2009) examined the AUC of the MHI‐5 for various anxiety disorders and found that the MHI‐5 was an adequate screener for OCD. As OCD was not assessed in our study, we could not replicate these findings. Of note, also when assessed as a single disorder without comorbid disorders, the AUC was acceptable to excellent.

The optimal cut‐off value on the MHI‐5 that was associated with the highest sensitivity and highest specificity for an anxiety disorder was ≤76. This finding is difficult to compare with other studies as other studies did not calculate cut‐off values, or they used a different definition of anxiety disorder than we did. Lower cut‐off values were previously found for an anxiety disorder in a regional study (<70 in Rumpf et al., 2001) and for both panic disorder and GAD in a general population study (<70 and <62, respectively in Cuijpers et al., 2009). However, the AUC for any anxiety disorder in the present study was associated with a similar sensitivity measure compared to those in the two previously mentioned studies.

For the specific anxiety disorders the optimal cut‐off score on the MHI‐5 for any anxiety disorder also resulted in acceptable sensitivity and specificity values (i.e., ≥0.70). As in previous studies (Berwick et al., 1991; Cuijpers et al., 2009; Rumpf et al., 2001), the MHI‐5 was less accurate in detecting anxiety disorders compared to mood disorders. This is to be expected given that three of the five items that form the MHI‐5 refer to symptoms in the diagnostic criteria for mood disorders, while two refer to anxiety symptoms.

#### 4.3.3 Mood or anxiety disorders

Similar to the AUC for mood disorders and anxiety disorders, the AUC for any mood or anxiety disorder was excellent. The optimal cut‐off score on the MHI‐5 for any mood or anxiety disorder was similar to that for any anxiety disorder (i.e., ≤76). These findings are relatively new as when previous studies looked at the validity of the MHI‐5 for screening mood and anxiety disorders, they usually did not report its validity for screening mood or anxiety disorders as one group.

#### 4.3.4 Multiple mood or anxiety disorders

The present study showed that the MHI‐5 is also an adequate instrument for detecting people with multiple mood or anxiety disorders, which may be associated with more severe functional limitations. The optimal cut‐off score on the MHI‐5 for multiple mood or anxiety disorders was ≤72 according to the Youden method, which was associated with a sensitivity of 0.92 and a specificity of 0.83. This complements previous research because to our knowledge the validity of the MHI‐5 for screening multiple mood or anxiety disorders has not previously been investigated.

The MHI‐5 is an excellent instrument for detecting people with frequent comorbidity patterns (AUC's varied between 0.89 and 0.96). Moreover, the cut‐off value for at least two mood or anxiety disorders resulted in excellent sensitivity and specificity values for the specific comorbidity disorder patterns ​(ranging from 0.82 to 0.97 and from 0.82 to 0.83, respectively). Only Cuijpers et al. (2009) reported on the ability of the MHI‐5 to detect people with a comorbid disorder (i.e., MDD and dysthymia), they found somewhat lower accuracy values.

## 5 CONCLUSION AND IMPLICATIONS

The MHI‐5 is a good measure for identifying individuals in the general population who are at high risk for a mood disorder, an anxiety disorder, or any mood or anxiety disorder in the past month, when clinical interviews are too time‐consuming. The MHI‐5 performed best in detecting mood disorders. Further research could focus on the abilities of the MHI‐5 to indicate the severity of a disorder, and to assess whether the MHI‐5 could also indicate functional limitations when having a depression or anxiety disorder, as we were only able to assess the number of disorders.

## AUTHOR CONTRIBUTIONS

**Margreet Ten Have**: Conceptualization, writing ‐ original draft, writing ‐ review & editing, formal analysis, funding acquisition, resources, methodology. **Marja J. H. Van Bon‐Martens**: Conceptualization, writing ‐ review & editing, methodology. **Frederiek Schouten**: Conceptualization, writing ‐ review & editing, formal analysis, data curation. **Saskia van Dorsselaer**: Conceptualization, writing ‐ review & editing, data curation. **Laura Shields‐Zeeman**: Conceptualization, writing ‐ review & editing. **Annemarie I. Luik**: Conceptualization, writing ‐ review & editing, supervision.

## CONFLICT OF INTEREST STATEMENT

The authors have no conflict of interest.

## Supporting information

Table S1

## Data Availability

The data on which this manuscript is based are not publicly available. However, data from NEMESIS‐3 are available upon request. The Dutch ministry of health financed the data and the agreement is that these data can be used freely under certain restrictions and always under supervision of the Principal Investigator (PI) of the study. Thus, some access restrictions do apply to the data. The PI of the study is first author of this paper and can at all times be contacted to request data. At any time, researchers can contact the PI of NEMESIS‐3 and submit a research plan, describing its background, research questions, variables to be used in the analyses, and an outline of the analyses. If a request for data sharing is approved, a written agreement will be signed stating that the data will only be used for addressing the agreed research questions described and not for other purposes.
