# Supplementary material for: Validity of the five‐item mental health inventory for screening current mood and anxiety disorders in the general population
Source: Int J Methods Psychiatr Res. 2024 Jul 2;33(3):e2030. doi: 10.1002/mpr.2030 (PMC11219508; doi:10.1002/mpr.2030)
Supplement: Supplementary file 1 — Table S1 [file MPR-33-e2030-s001.docx]

Supplementary Table 1 MHI-5 scores in people with 12 month DSM-5 mood and anxiety disorders: means and standard deviations, AUC statistics with 95% Confidence Intervals (CI)

|  |  | | MHI-5 | | | |
| --- | --- | --- | --- | --- | --- | --- |
|  | n | % | M | S.D. | AUC | 95% CI |
| 12-month prevalence rates |  |  |  |  |  |  |
|  |  |  |  |  |  |  |
| Any mood disorder | 550 | 8.89 | 65.48 | 18.66 | 0.83 | 0.81-0.85 |
| Major depressive disorder | 476 | 7.69 | 65.69 | 18.52 | 0.82 | 0.80-0.84 |
| Persistent depressive disorder | 191 | 3.09 | 59.37 | 19.87 | 0.86 | 0.83-0.89 |
| Bipolar disorder | 67 | 1.08 | 64.12 | 19.26 | 0.81 | 0.76-0.86 |
| Single disorders |  |  |  |  |  |  |
| Any mood disorder only | 329 | 5.32 | 70.13 | 16.93 | 0.76 | 0.74-0.79 |
| MDD only | 194 | 3.14 | 72.99 | 15.82 | 0.73 | 0.69-0.76 |
|  |  |  |  |  |  |  |
| Any anxiety disorder^1^ | 564 | 9.12 | 68.41 | 18.88 | 0.78 | 0.76-0.80 |
| Panic disorder | 120 | 1.94 | 65.83 | 21.01 | 0.77 | 0.72-0.82 |
| Agoraphobia | 109 | 1.76 | 62.83 | 19.85 | 0.83 | 0.79-0.87 |
| Social phobia | 315 | 5.09 | 68.46 | 18.91 | 0.77 | 0.74-0.80 |
| Generalized anxiety disorder | 219 | 3.54 | 60.80 | 19.26 | 0.86 | 0.83-0.88 |
| Single disorders |  |  |  |  |  |  |
| Any anxiety disorder only^1^ | 343 | 5.54 | 74.75 | 15.84 | 0.70 | 0.67-0.72 |
| SO only | 158 | 2.55 | 78.58 | 14.21 | 0.63 | 0.59-0.68 |
| GAD only | 59 | 0.95 | 70.71 | 15.62 | 0.76 | 0.70-0.82 |
|  |  |  |  |  |  |  |
| Any mood or anxiety disorder^1^ | 893 | 14.44 | 69.04 | 18.19 | 0.80 | 0.78-0.81 |
|  |  |  |  |  |  |  |
| At least two mood or anxiety disorders^1^ | 376 | 6.08 | 60.97 | 18.19 | 0.87 | 0.86-0.89 |
|  |  |  |  |  |  |  |
| Comorbid disorders |  |  |  |  |  |  |
| MDD + PDD | 184 | 2.97 | 59.17 | 19.72 | 0.86 | 0.83-0.89 |
| MDD + GAD | 108 | 1.75 | 55.52 | 20.26 | 0.89 | 0.86-0.92 |
| SO + AG | 61 | 0.99 | 54.75 | 18.84 | 0.91 | 0.88-0.94 |
| SO + Specific phobia | 88 | 1.42 | 61.73 | 19.18 | 0.84 | 0.80-0.88 |
| GAD + PDD | 56 | 0.91 | 49.36 | 20.60 | 0.92 | 0.89-0.96 |
| GAD + SO | 71 | 1.15 | 53.52 | 17.77 | 0.92 | 0.89-0.95 |
|  |  |  |  |  |  |  |

^1^: Specific phobia was not included in the category of anxiety disorders as the AUC was poor (AUC: 0.65, 95% Confidence Interval (CI): 0.62-0.68).
